# Refugee-led socio-spatial organization in Al Baqa’a camp, Jordan

**DOI:** 10.1186/s40410-021-00145-y

**Published:** 2022-01-12

**Authors:** Rania Aburamadan

**Affiliations:** The Department of Architecture, Applied Science Private University, Al Arab St. 21, Amman, 11931 Jordan

**Keywords:** Refugee, Refugee shelters, Shelter design, Socio-cultural, Al Baqa’a camp, Jordan

## Abstract

The increase in refugee numbers is an increasingly important concern globally. Many countries in different regions have been accommodating refugees by providing temporary shelters made from ineffective and inadequate materials to provide thermal comfort for refugees. However, the shelters provided are often inadequate solutions for shelter and neglect the social and cultural diversity of the refugees. Socio-cultural norms, practices and values are rarely considered in the design of shelters and this has an adverse impact on how refugees live in these spaces. Using insights from the Al Baqa’a refugee camp in Jordan as a case study, this paper uses a mixed-method approach to explore how the challenges of inadequate shelter has consequently led refugees to self-organize and create new socio-cultural spaces to adapt to the place. The findings suggest that historically, Al Baqa’a camp has reorganized by users due to social needs and climate challenges. When the camp was created in 1967, the inadequacy of the housing and infrastructure to provide comfort influenced refugees to self-organize and create adaptive spaces of comfort. However, over the decades, these spaces have evolved into spaces of enterprise, belonging and memory of their homeland. Therefore, this paper argues that refugee shelter design should have an integrated consideration of the climatic elements and the social and cultural aspects of refugees. The paper concludes with lessons learned drawn from the evidence to act as guideline for the consideration of official humanitarian organizations in other camps and local communities.

## Introduction

A myriad of situations are leading to the unprecedented growth of the refugee’s crisis. The global landscaping is facing present challenges such as the pandemic, growing effects of climate change, humanitarian emergencies and increasing political instability. The United Nations High Commissioner for Refugees (UNHCR) has reported (UNHCR 2020) a rapid increase in the global population of forcibly displaced people from 43.3 to 79.5 million between 2009 and 2019. The majority of this increase occurred between 2012 and 2015 due to the Syrian conflict in 2011. Furthermore, conflicts in Iraq, Yemen, the Democratic Republic of the Congo (DRC) and South Sudan have contributed to this displacement, as well as the massive flow of Rohingya refugees from Myanmar to Bangladesh in 2017 (UNHCR 2019). Furthermore, hardships and displacement has multiplied amid the COVID-19 pandemic (UNHCR 2020). The uniqueness of the lived experiences of refugees in refugee camps has given way to an increasing global discourse that is investigating the improvement of refugees and the camp space (Agier 2002, 2008; Alnsour and Meaton 2014; Ashmore et al. 2003; Brun 2001; Hart et al. 2018; Herz 2012). Jordan has a historical relationship with refugees and has been housing refugees since 1984 up till present. The main stakeholders of the refugee camps in Jordan are refugees, governments and non-government organizations (NGOs) (UNHCR 2019).

In a crisis, humanitarian agencies tend to first provide tents as an emergency “temporary” solution requiring a provisional accommodation for a limited time. This is the case for some refugee camps setup for example in response to natural disasters such as the case in Chile or Japan. Various intermediate conditions such as the prolonging of crisis leads to socio-spatial evolutions and requirements. Consequently, there is no universal agreement of the actual conceptualisation of the refugee camp space (Aburamadan et al. 2020). It has been described as temporary, transient, city-camps, semi-permanent, spaces occurring between the two concepts of “permanent” VS “temporary” solutions created “between war and city” (Agier 2002). These conceptualisations shape the lived experiences of refugees and how they interact with the camp space (Stevenson and Sutton 2011). The solutions for establishing dwellings by UNHCR have considered the basic design and function of refugee shelters as an unintended consequence of a decision made on purely functional and financial grounds (Corsellis and Vitale 2005, 2008; Manfield et al. 2004). Therefore, a flexible strategic approach in addressing refugee camps is required. This constant change of socio-spatial stages reinforces the suitability of architecture and urban planning in terms of contributing to the understanding of the solution and way forward in this regard. This perhaps contradicts the one-size-fits-all approach currently underpinned in international guidelines and documents leading towards the favouring of temporary structures. As a result, refugees can end up living in refugee camps for many years in temporary shelters made from lightweight materials that are ineffective against harsh climatic conditions (Aburamadan et al. 2020).

**Fig. 1:**
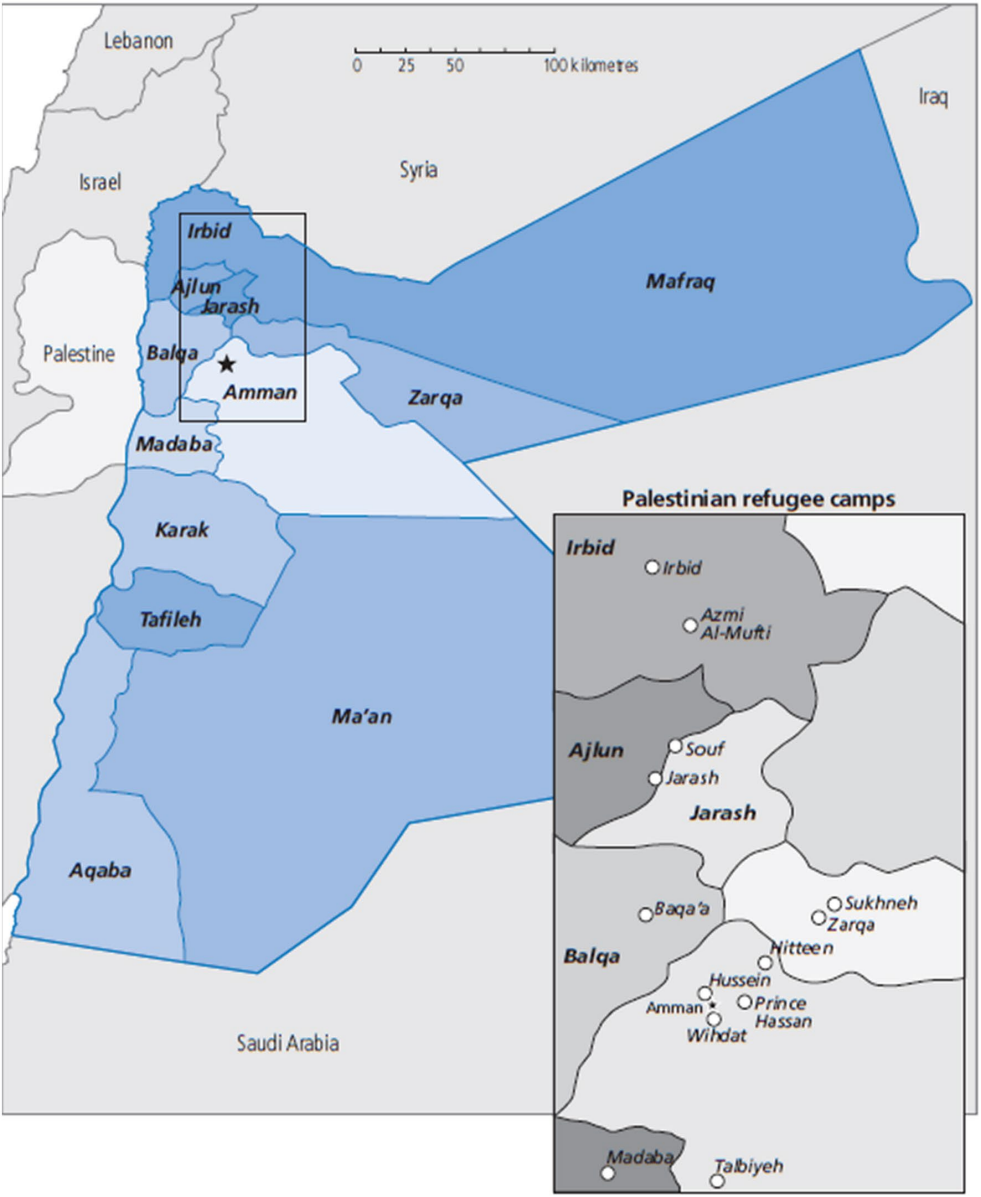
Map of Jordan with Palestinian refugee camps (UNRWA 2020)

As a nation, Jordan has become home to an increasing flow of refugees thereby demanding a significant number of shelters and services. The refugee camps in Jordan are setup in a hot-dry climate which is made even harsher by climate change and related desertification. Almost 2 million Palestine refugees and a large number of other Palestinians were displaced as a result of the 1967 war and subsequent hostilities and relocated to Jordan. Some of these refugees are accommodated in Jordan’s refugee camps (see Fig. 1) whilst others live alongside other Jordanians in cities, towns and villages (UNWRA 2013). As a country, Jordan is located in the Middle East and North Africa region. The region differs from hot dry climates in North Africa, Jordan and Iraq except for the highland of Jordan Valley to the moderate climates in Palestinian, Syria and Lebanon. Jordan sits at the crossroads of Asia, Africa and Europe and is divided into three geographical areas; Rift Valley in the west, the highland in the center and the desert in the east. Two-thirds of Jordan is located in the desert. The climate of Jordan is hot and dry especially in the eastern and northwestern areas. The variation in temperature is dramatic between summer and winter as well as day and night. The greater contrast between day and night or summer and winter is the major concern in providing comfort inside dwellings due to the high cost of energy for cooling and heating (Johansson et al. 2009). Thus, the dominant concern is the winter season in the desert zone and the summer season in the highlands zone.

This paper examines the Al Baqa’a refugee camp as a case study. This camp is the largest in Jordan and is located approximately 20 km North of Amman, the Capital city of Jordan. As a camp it is one of the most longest standing camps having been setup in 1968 to accommodate Palestine refugees and displaced people who left the West Bank and Gaza Strip as a result of the 1967 Arab–Israeli war (UNRWA 2020). The camp has evolved considerably since its conception. The camp started as a defined plot of land with relief tents and has developed through UNRWA intervention and refugee-led interventions into a thriving camp with existing streets, roads, enterprise and built infrastructure. Today, what exists can be perceived as a hybrid of temporariness and permanence, a type of informal city (Aburamadan et al. 2020). The harshness of the climate in Jordan has contributed to the settling of refugees in the Al Baqa’a camp. This was particularly the case at the conception of the camp as refuges were living in severely inadequate housing. Over the decades, this housing has changed and influenced how refugees have adapted and created spaces that function for them. Furthermore, the climatic conditions played a significant role in shaping the socio-cultural elements of refugees’ lives. Therefore, this paper aims to explore how the challenges of inadequate shelter infrastructure has consequently led refugees to self-organize, self-adapt and create new socio-cultural spaces.

This paper is structured by firstly presenting the theoretical framework by exploring the design of refugee shelters and the socio-spatial strategies of refugees through self-organization in “Theoretical framework” Section. This is followed by “Case study: the Al Baqa’a refugee camp, Jordan” Section, the outline of the context of the case study, the Al Baqa’a camp in Jordan and chosen methodology. “Refugees’ everyday experiences living in the Al Baqa’a camp” Section discusses the data findings. The lessons learned from the evidence is highlighted in “Refugees’ socio-spatial practices and self-organization” Section with concluding thoughts in “Emerging lessons learned” Section.

## Theoretical framework

### Refugee shelters designed by humanitarian organizations

**Fig. 2:**
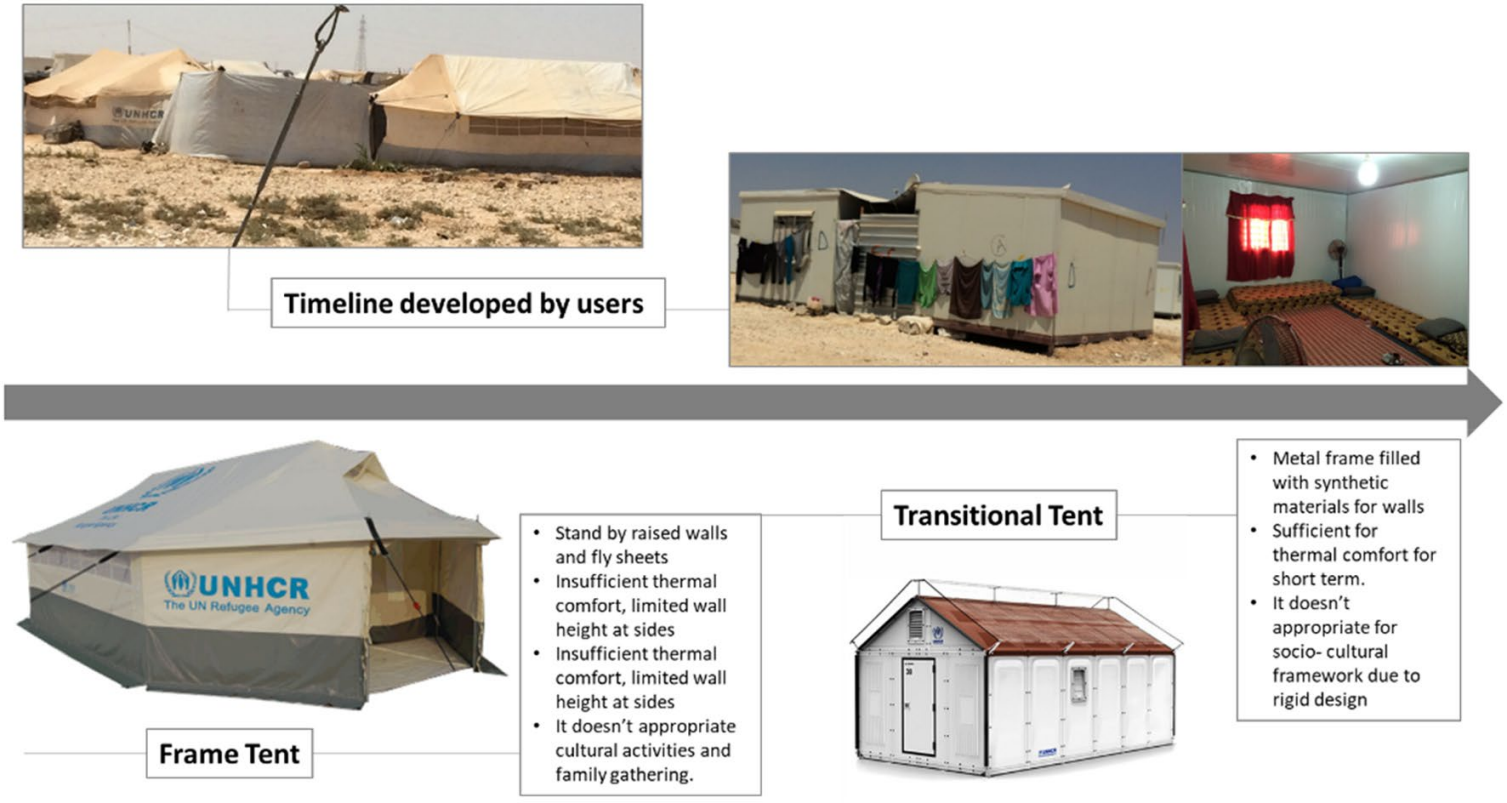
Humanitarian organized shelters and user adapted shelters

Immediate shelters provided by humanitarian agencies are often standard tents with different characteristics and their dimensions are based on the number of occupants, materials and shape. Over the last few decades, the type of shelter provided by humanitarian organizations has evolved considerably parallel with the shifts in aid landscape. In the 1960s, humanitarian shelter response was in its early stages and therefore shelter tended to take the form of tarpaulins, tents and makeshift shelters created from temporary found materials. Efforts have been made as time has moved on, to build more permanent housing for refugees. However, in most cases this move has seen resistance from Humanitarian organizations due to issues of cost and resources as well as the perceived nature of permanence within a refugee camp (Aburamadan et al. 2020). Transitional shelters (Fig. 2) are therefore preferred as an alternative to the temporariness of the tent and the permanence of the built housing. These transitional tents are often made to be erected with ease and made from materials which can be re-used. An example of this is the IKEA shelter created in association with the Swedish furniture company, IKEA and the United Nations to provide refugee shelter at a global scale. The IKEA foundation has reported that refugees can use the shelters for 6 months before the effects of climate conditions such as sun, wind, humidity and rain are experienced. Therefore, despite the convenience and cost-saving elements of providing these transitional shelters, thermal comfort is guaranteed for a limited time and the shelter does not consider the socio-cultural aspects of the refugees. Refugees adapt the humanitarian organized shelters according to the needs and social practices. Figure 2 illustrates how refugees appropriate the humanitarian shelters to create individual, in-between spaces to accommodate the practices within their households. These self-adaptations are seen with each stage of the tent process, from the frame tent into the transitional tent.

**Fig. 3:**
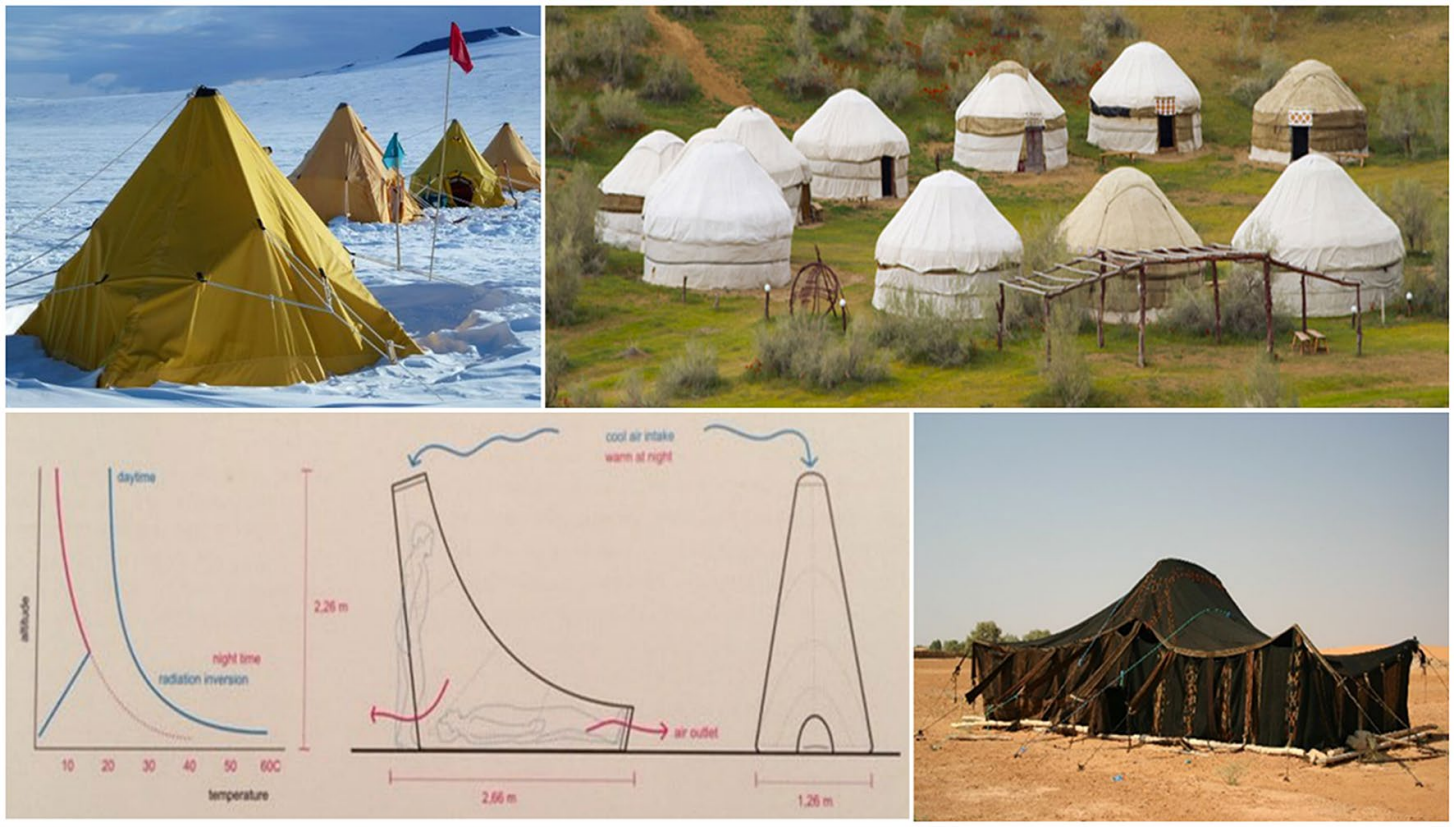
Examples of traditional refugee tents on top and bottom left, the DesertSeal by ESA and bottom right the Tuareg tent by IFRC

The comparison of the UNHCR tent with a traditional tent is challenging. Refugees are often found to be culturally and environmentally better adapted to the traditional tent given the use of natural materials and the efficiency of the traditional design to provide comfort. Because of this, many types of traditional tent are presented in different regions and climates such as the Koryak and Chukchi tents in northern Asia, the Scott tent, Yurts as shown in the figure below and others, but most of them share the same conclusion of adapting to climates (Manfield 2000). Therefore, numerous agencies use the elements and functionalities of a traditional tent in the creation of a new tent. An example of this is the Tuareg tent developed by the IFRC and the DesertSeal developed by the European Space Agency (ESA). These two examples as shown in Fig. 3, were created to mimic the traditional tent with considerations of the hot-dry climate conditions. Research has evidenced that refugees use appropriate traditional tents adaptively to the surroundings and environment due to the development practices of their generations (Attia 2014). In such a way this establishes and maintains the use of familiar materials and worker skills. Refugees create and adapt because they are living in this environment and utilize generational information on how to build this and what specific materials to use. Urban design interventions in refugee camps play a crucial role in achieving a comfortable balance between these factors and human activities (Attia 2014). Therefore, the consideration of factors such as the layout of refugee dwellings, insulating building materials, good quality design and structure and urban organization should be considered at an early stage of the design process of refugee shelters (Corsellis and Vitale 2005). Evidence suggests impacts such as increasing temperatures and increased incidence of severe storms are being experienced more acutely in refugee camps. Refugees are often found to be living in temporary shelters inadequate for thermal comfort and subject to extreme climate conditions. However, there is a paucity in scholarly discourse despite the relevance of practice of architecture, design and urban planning in achieving thermal comfort and quality of life for refugees in refugee camps. A systematic literature review conducted by Albadra et al. (2017) demonstrated that the literature on the design of post-disaster relief shelters limited, with fewer than sixty publications in the past 4 decades. Few publications focused on the thermal performance or thermal comfort in refugee shelters despite evidence of extreme discomfort (Albadra et al. 2017). The findings revealed that most families in hot arid climates within their study reported unbearable discomfort in their shelters from extreme heat during the day and extreme cold at night. A study on self-built shelters in Bangladesh by Klansek et al. 2020 uncovered the need for going beyond mass-produced housing as solutions, to housing that incorporates key aspects of socio-cultural living, privacy, security and thermal comfort. Although the study suggests that self-built adaptations and self-built accommodation has the potential to enhance living conditions and occupant satisfaction. Refugees living in camps often do not have access to electricity and the main cooling strategy at building level is natural ventilation. A social survey by Paszkiewicz and Fosas (2019) highlighted that coping mechanisms against heat were mainly to shower, to pour water onto themselves with their clothes on and to spray water on the floor. These coping strategies interrupted any day to day activities. The inadequacy of the shelter environment to cope with the heat influenced how refugees commune together and find comfort.

### Refugee camps as socio-cultural spaces and places

**Fig. 4:**
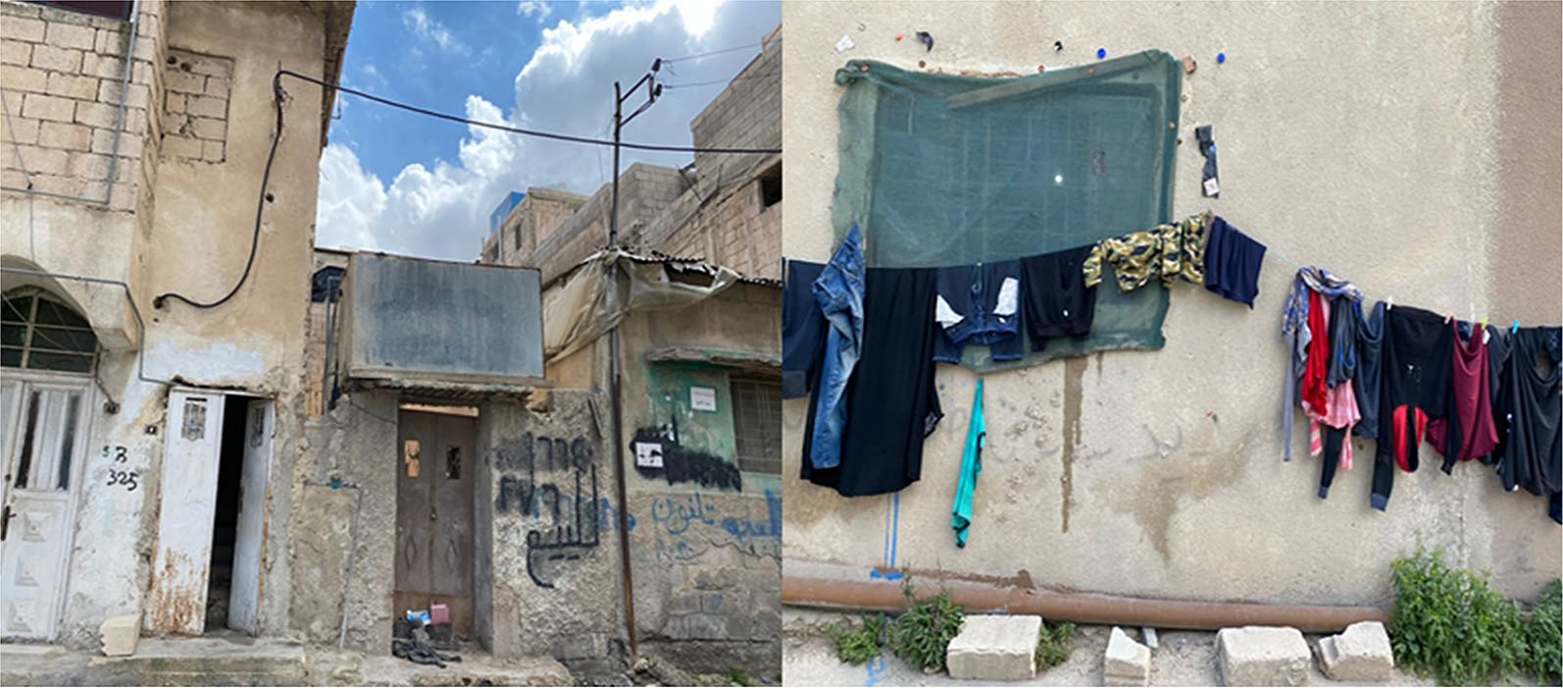
Refugee camps as socio-cultural spaces and places (Source: Author 2021)

Very few of the studies mentioned in the above sections have explored the connection between the socio-spatial and cultural nature of refugee camps and the technicality of achieving thermal comfort in the shelters. To achieve a humanitarian shelter that respects the socio-cultural norms of refugees while offering a comfortable and energy efficient shelter requires an acknowledgment of the socio-spatial places, spaces and practices within refugee camps. The concept of place is often understood in academic discourse as spaces that allow attachment and meaning to the people who use those spaces (Lombard 2014; Sampson and Gifford 2010; Brown et al. 2012). Using this concept in this study is useful in providing an additional layer of understanding to how refugees settle in refugee camps as well as revealing the elements of power to transform a space to a place (Fábos and Kibreab 2009; Mah and Rivers 2016; Lombard 2014). Refugees in camps are rarely empowered to act as citizens and appropriators of the camp. International and local humanitarian organizations are primarily concerned with the protection and survival of inhabitants (Stevenson and Sutton 2011). Kennedy et al. (2008), explained that part of the challenge is that the handbook of the UNHCR frames refugee camps as an isolated site excluded from interaction and integration with the local community it surrounds. Therefore, the space inhabited by refugees often mirrors the lack of consideration for the social construct allowed by a consolidated urban fabric (Hart et al. 2018; Schmeidl 2002; Paszkiewicz and Fosas 2019). However, refugees in camps continue to show their willingness to transform their space (Fig. 4) (Albadra et al. 2021) by making a place that bears a resemblance to a permanent built environment (Agier 2002). Focusing on the discourse on place foreground the everyday social nature of refugee camps, thus moving away from the perspective that views these camps as constant emergency states of temporariness (Lombard 2014; Certeau et al. 1998). This gives attention to the ordinary sociality of refugee camps drawing to the importance of people as autonomous actors who creatively engage with, and shape, their surroundings (Albadra et al. 2021). This leads to an increasing level of self-organization within the camps space as a form of adaptation. The introduction of self-organization in the refugee space shifts away from space as a passive component and towards the production of social relations (Brown et al. 2012; Livingston et al. 2008). The attachment of people to a place has a deep association that is often linked to an individual’s sense of belonging, identity and security (Scannell and Gifford 2010). This is described as “homemaking” by Hart et al. (2018), the actions and aspirations of camp residents to transform their dwellings with a sense of home (Hart et al. 2018; Paszkiewicz and Fosas 2019). Modifications to the shelter to facilitate social activities has been a large part of the socio-spatial transformation of the camp to suit their needs towards thermal comfort and express their values (Knox and Mayer 2013).

There are two refugee camps that display the embedding of socio-spatial qualities within the refugee camp space. Naher El Bard and Al Baqa’a are located in the same region, same period of time and this is the same for Palestinians which is a justification to emphasize the power of socio-spatial aspects although there exist some differences in climatic issues. Firstly, the Nahr El Bared Camp in Lebanon attempts to include the social practices of refugees in the re-construction and master planning process and secondly, refugees in the Al Za’atari Camp, Jordan have shown levels of socio-spatial adaptation to achieve thermal comfort and ultimately provide a sense of belonging within the camp.

**Fig. 5:**
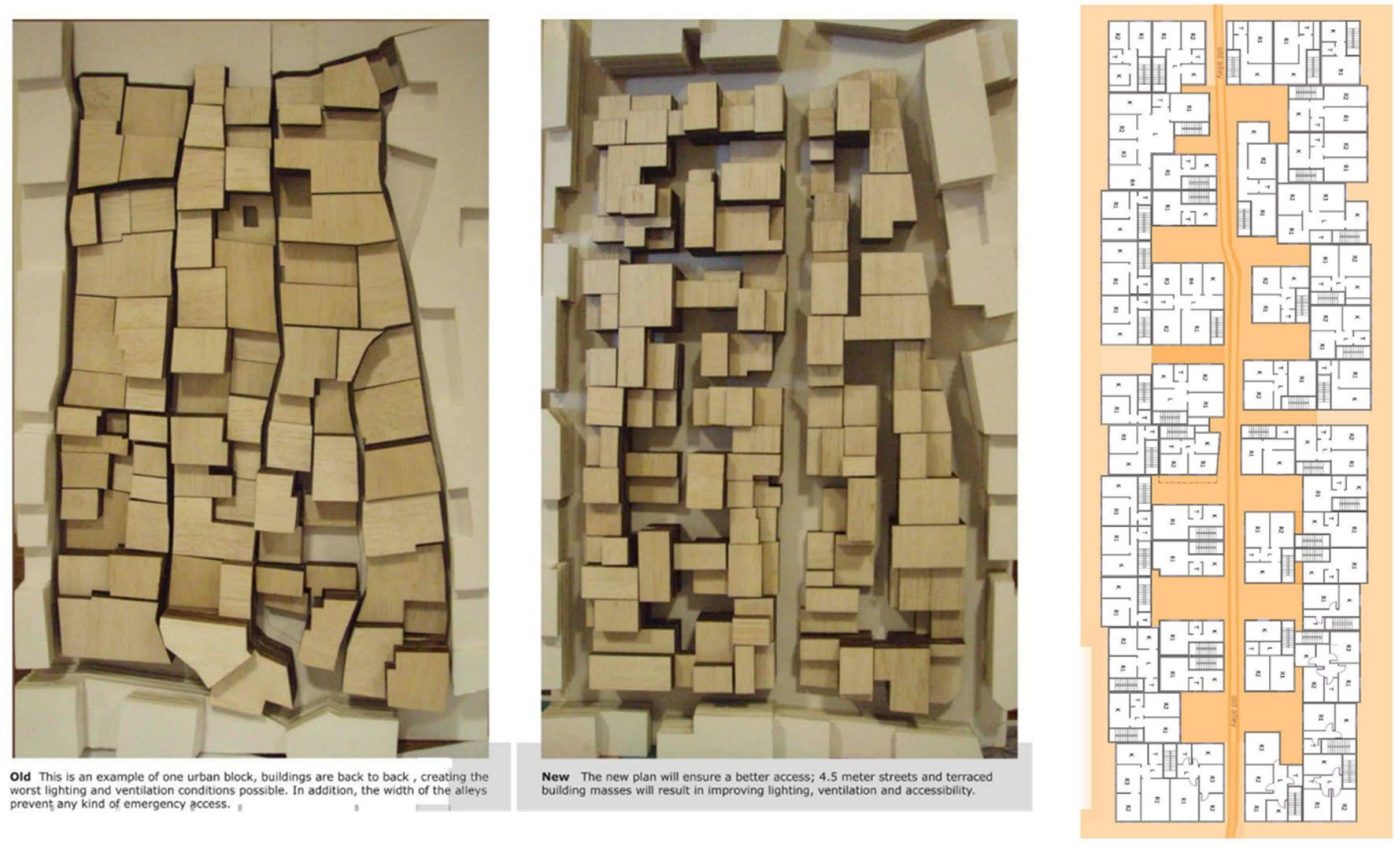
Nahr El Bared Refugee Camp (Source: Author 2016)

The Nahr El Bared camp is in the north of Lebanon and it is considered to be the second largest camp in Lebanon with a total population of 30,000 Palestine refugees. The camp was established in 1949. The continuous demand on housing associated with socio-economic growth and limited space has turned the camp into a highly dense urban fabric with high population density. Confrontation started in May 2007 between Fatah al-Islam and the Lebanese Army with devastating consequences on Nahr el Bared community and resulted in a complete destruction of the camp and the displacement of almost 30,000 civilians. The rebuilding of the camp after the destruction initially took the form of community workshops and increased dialogue with the local community-based organisations and NGO’s who played a leading role in formulating and endorsing the guidelines. It was agreed that the focus was primarily on rebuilding the entire community through respect and sensitivity to socio-cultural values (Fig. 5). The preservation of social spaces and practices was essential to the design of the Master Plan as well as ensuring that there was accommodation for economic activities that can sustain the refugees. The new proposed masterplan of the refugee camp ensured better lighting, accessibility, wider streets and terraced building masses.

**Fig. 6:**
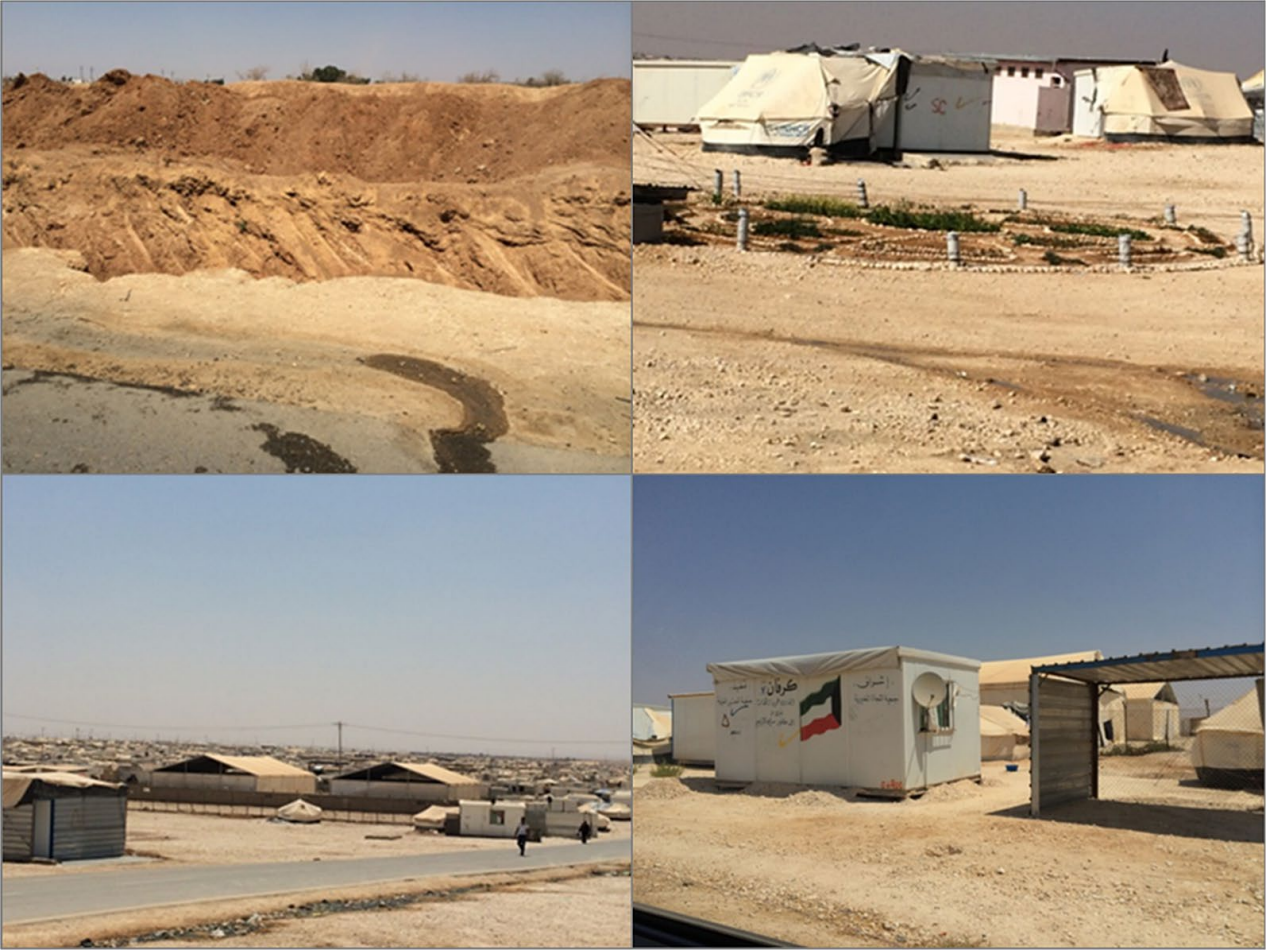
Informal layout in the Al Za’atari camp in Jordan (Source: Author 2013–2015)

**Fig. 7:**
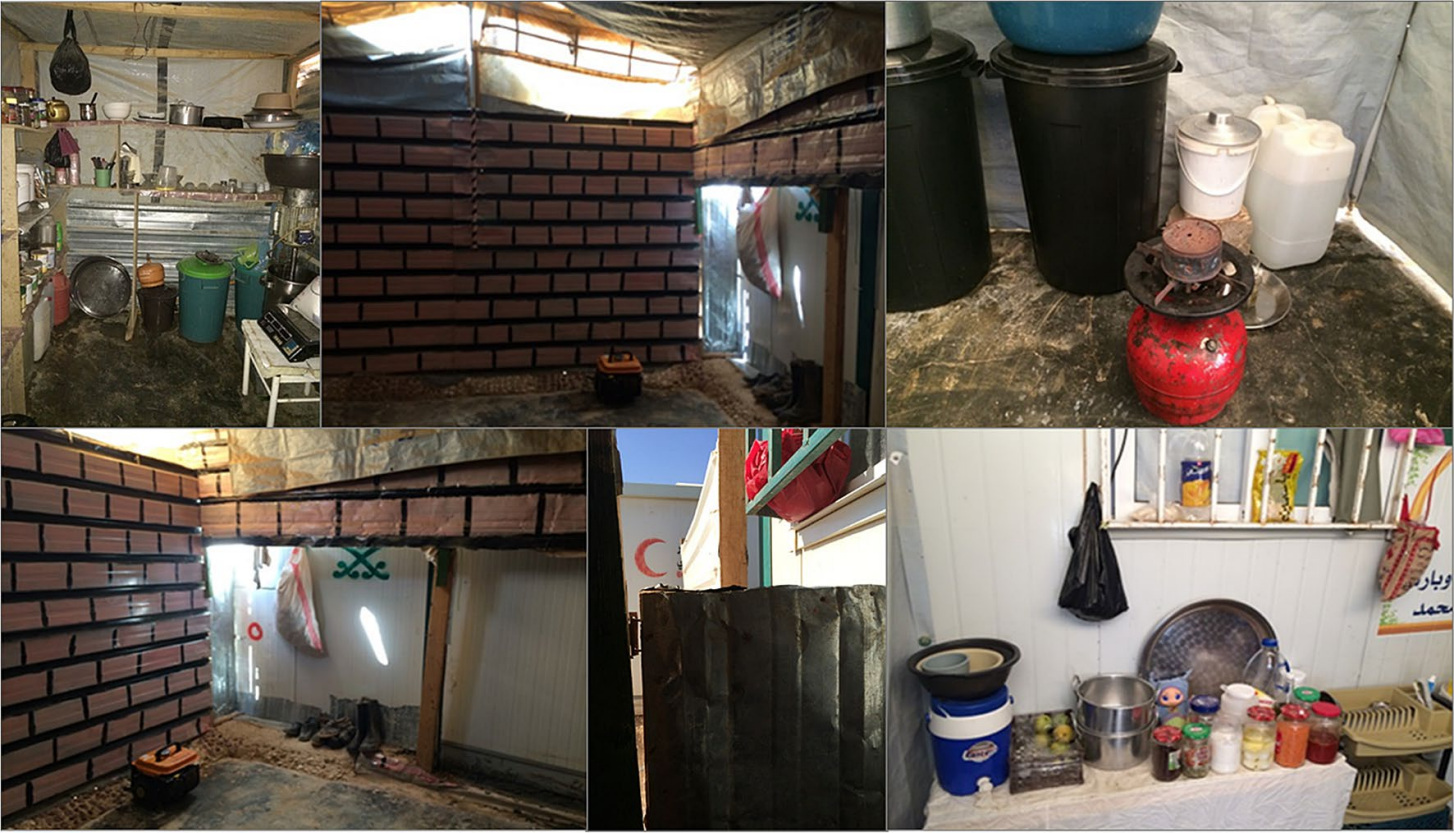
Refugee created semi-outdoor socio-cultural spaces in the Al Za’atari camp in Jordan (Source: Author 2013–2015)

The Al Za’atari camp in Jordan is located in the desert zone, 10 km east of the Mafreq Governorate and it is largely surrounded by rural areas. The camp environment has a topography which can be described as a desert which is often flat and sandy. Refugees living there experience extreme conditions of hot summers and cold winters. The Al Za’atari camp become the second largest camp in the world accommodating a large number of Syrian refugees (Ledwith 2014). The camp initially began as a grid organization like most refugee camps as per humanitarian organization standards. However, over a longer time period, the camp is evolving through social organization into a more informal layout (Fig. 6). Refugees made shelter modifications as shown in Fig. 7. The space transformed according to use and began to adopt a tradition in Syrian houses where families meet each other every day for singing or just gathering to share memories of their homeland. A study on participatory design in refugee camps by Albadra et al. 2021, conducted “design and adapt your own home” participatory activities in the Al Za’atari camp. Proposed housing designs from the participants included shelters with larger space and several more rooms for different tasks and social practices.

## Case study: the Al Baqa’a refugee camp, Jordan

The Al Baqa’a camp has been established on a very fertile agricultural area in Jordan which historically used to produce a quarter of the food basket of Jordan. The camp was formed in 1968 as one of six temporary and emergency camps intended to shelter Palestine refugees and displaced persons who migrated from the West Bank and Gaza Strip due to the 1967 Arab–Israeli war (UNRWA 2020). Between June 1967 and February 1968, the resident refugees and displaced people were housed in temporary camps in the Jordan valley. However, they had to be removed when military operations escalated in the area between the Zionist occupation army soldiers and the Palestinian guerrillas. The Al Baqa’a camp was set up initially as a large camp with almost 5000 tents for 26,000 refugees and these numbers have continued to increase although they are not linear and can fluctuate depending on various factors such as the effects of war and political instability (Lintelo et al. 2018).

A semi-ethnographic approach was considered the most suitable for this study. It is based on the research philosophy of interpretivism as an epistemological paradigm. This paradigm aligns with the author’s belief of foregrounding the perspective of the refugees and their socio-cultural practices. The study allows a comprehensive examination of a dataset of evidence and the considerable time spent in the field of the Al Baqa’a camp. Therefore, a single case study strategy was chosen as the methodology, in order to allow for an in depth understanding of the intertwined and complex socio-economic dynamics happening inside the camp and across different stakeholders. This can also be perceived as a limitation of this study as the Al Baqa’a has unique characteristics that cannot be considered an average, critical or exceptional case study (Flyvbjerg 2006). The motivation beyond the construction of Al Baqa’a influences the transferability of findings and conclusions (Aburamadan et al. 2020). The selection of the Al Baqa’a camp in Jordan has been based on the rationale that this is the largest Palestinian camp in Jordan and one of the largest in the world. Refugee camps are setup for diverse reasons such as those with high degrees of predictability and temporariness (e.g., flooding), therefore this case cannot be considered equally relevant to other cases of refugee camps. The aim of this paper is to shares the lessons learned from this in-depth case study and contribute to the understanding of refugee camps and the socio-cultural impact. To do this, this study is based on direct observation pursued through 3 visits to the Al Baqa’a camp and a robust qualitative dataset of semi-structured interviews.

In-depth semi-structured interviews were conducted for this study on the Al Baqa’a camp during 3 visits to the camp. 24 in-depth semi-structured interviews were conducted with refugees and a further 40 in-depth semi-structured interviews with experts and professionals in the field (manufacturers, NGOs professionals, academics, researchers) were also administered. Questionnaires were also conducted with responses from 83 refugees. The interviews with refugees were purposed to shape the understanding of the refugees’ socio-cultural and spatial response to living in a refugee camp. The interviews with the experts allowed for the expert perspective and the verification of the official literature regarding the approach followed by NGOs and international organizations in designing and implementing camps. The interview questions were designed using gaps identified in the literature on refugee camps. Interviews and questionnaires with refugees considered the age, social status, gender and educational level and other attributes. They were conducted in Arabic by a female researcher, to allow for the wider participation of all genders, and where administered under the surveillance of the local police. The interviews and questionnaires were recorded and transcribed from Arabic and further translated into English for coding and identification of key-nodes. A thematic analysis of the interviews has been conducted to improve the possible knowledge of the related variables that influence the socio-spatial changes and improvements of refugees’ living standards.

## Refugees’ everyday experiences living in the Al Baqa’a camp

**Fig. 8:**
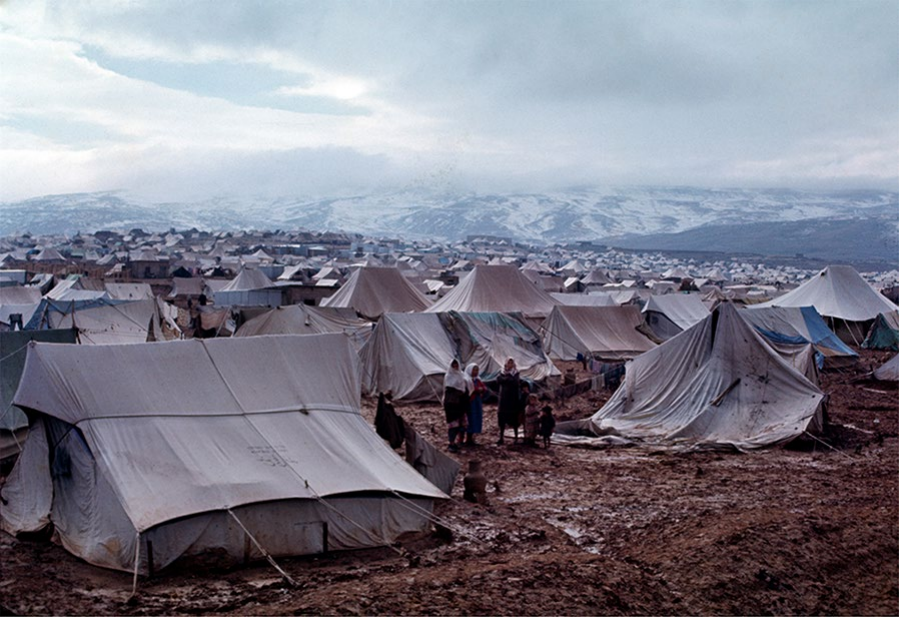
Overview of Al Baqa’a emergency camp © 1969 UNRWA Archive Photo by Munir Nasir

**Fig. 9:**
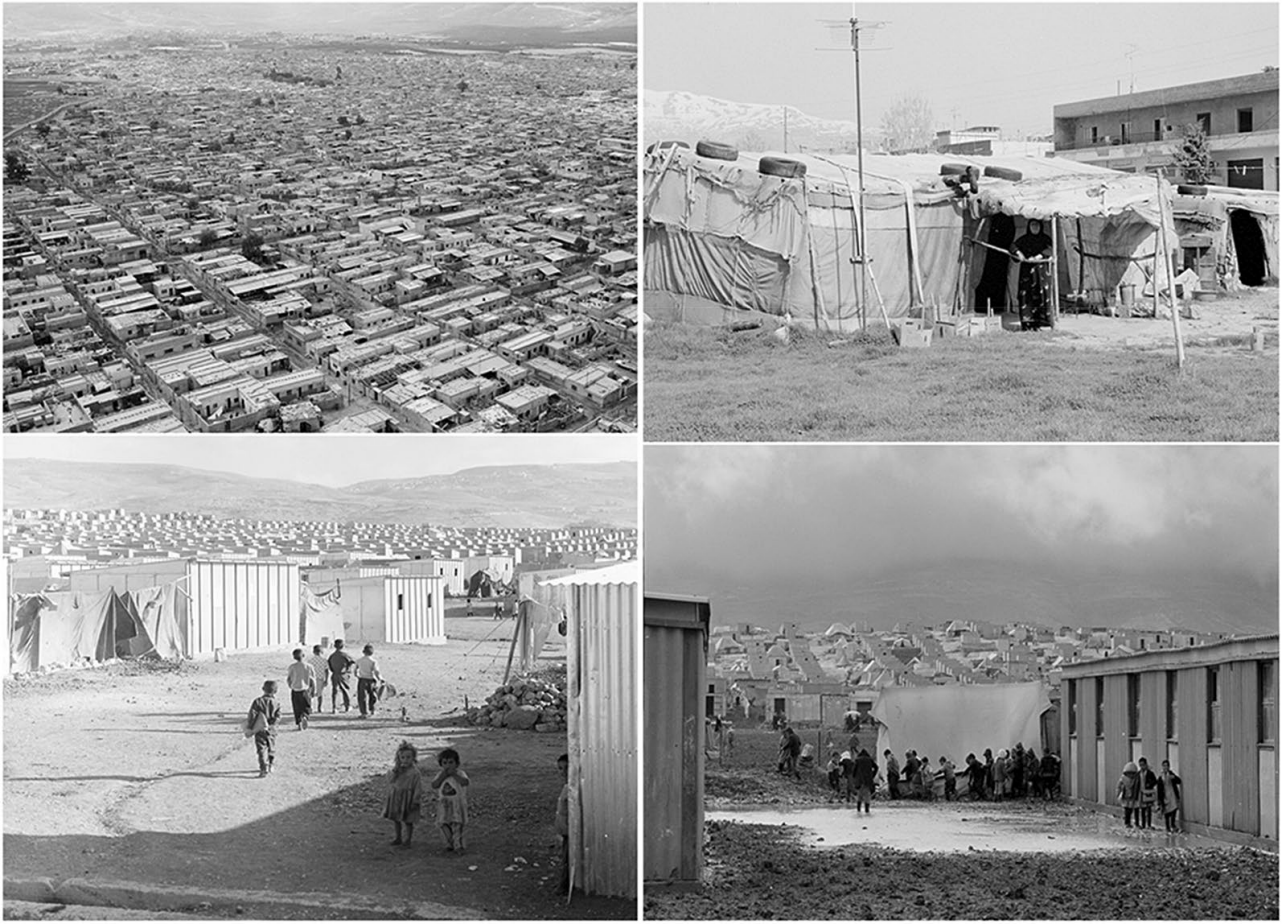
(Top and bottom-left) Al Baqa’a emergency camp tents have been replaced with prefabricated shelters. © 1987 UNRWA archive photo by J. Shammout © 1969 UNRWA archive photo by George Nehmeh; (top-right) Winter snow in the hills behind Al Baqa’a camp in Jordan. © 1967 UNRWA archive; (bottom-right) Palestine refugee children endure the harsh living conditions associated with winter in Al Baqa’a emergency camp, East Jordan, 1970. © 1970 UNRWA archive photo by Munir Naser

**Fig. 10:**
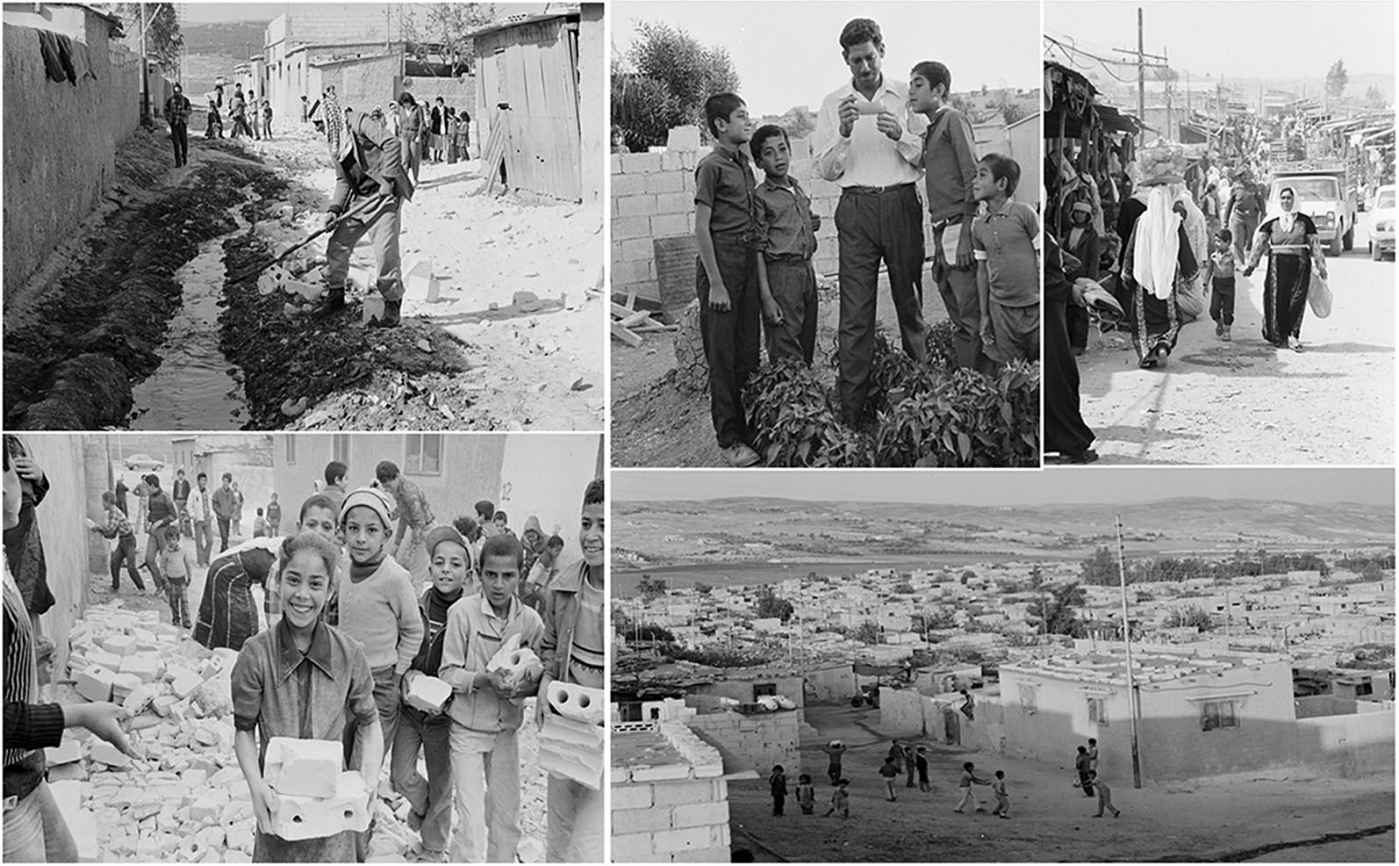
(Top and bottom left) self-help project to improve the environment et al. Baqa’a camp, Jordan. © 1984 UNRWA archive photo by Shaukat Hasan; UNRWA archive photographer unknown; (top-middle right) a family has managed to grow a garden in the overcrowded Al Baqa’a camp © 1971 UNRWA archive photographer unknown, (top-left) a busy market inside Al Baqa’a camp for Palestine refugees, east Jordan. © 1982 UNRWA archive photo by George Nehmeh and (bottom-right) Palestine refugee children play on the barren terrain which surrounds their prefabricated homes in Al Baqa’a camp. © 1983 UNRWA archive photographer unknown

The first form of shelter as an emergency measure in this camp was the relief tent. This relief tent varied according to the family size and there was no specific structure to the camp therefore refugees tended to be scattered according to their relatives (Fig. 8). The camp experiences extreme conditions of hot summers and cold winters. The figures below evidence the type of shelters the refugees were living in with the extreme conditions of winter snow in the hills behind Al Baqa’a camp. Between 1969 and 1971, the UNRWA replaced these tents with 8000 prefabricated shelters made from tin, wood and cardboard (Fig. 9). Despite the upgrade, the conditions, in addition to the absence of thermal insulating materials, resulted in the dwellings being very hot in summer and very cold in winter. Development of the UNRWA came with imposed specific regulations that determined how refugees could build and extend their homes. By the 1970s, a new generation was born in Al Baqa’a camp and more space was needed resulting in a refugee construction boom. This building was often led by refugees and determined by their practices, needs and desires, sometimes in violation of the UNRWA building regulations. Anything built beyond 100 m^2^ was considered an act of spatial violation (Maqusi 2017). Over the years, refugees began to demonstrate every day “normal” social practices such as cooking, gathering for meals and socializing, private tasks. The refugees created self-help projects designed by the community to improve their environment (Fig. 10). According to UNRWA, refugee camp inhabitants generally extended their standard homes to include a private toilet, kitchen, additional living spaces and a wall to enclose the plot and add privacy (Institute for Palestine Studies 2021). Horizontal spatial violations including outdoor thresholds and makeshift external stairs were built beyond the UNRWA plot demarcations. Social spaces were created by the refugees as a result of the camp improvements made. This included informal spaces for children to play outside during the hot weather, spaces to grow vegetables and also spaces of economy and enterprise (Fig. 10). The struggle for refugees to live “normally” led to the buying, selling, swapping and renting of shelters (Institute for Palestine Studies 2021), types of spatial economies that contest the formal enforcements of humanitarian and host policies (Maqusi 2017).

**Fig. 11:**
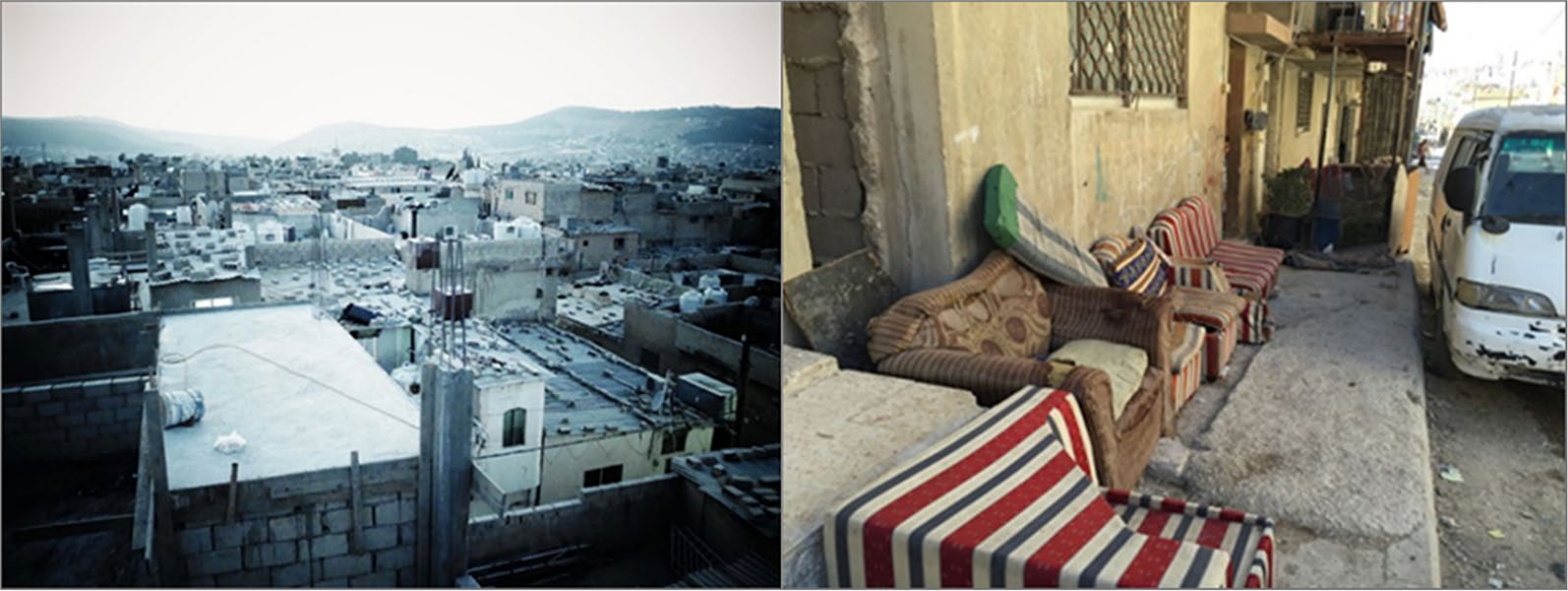
Refugee camps as socio-cultural spaces and places (Maqusi 2017)

**Fig. 12:**
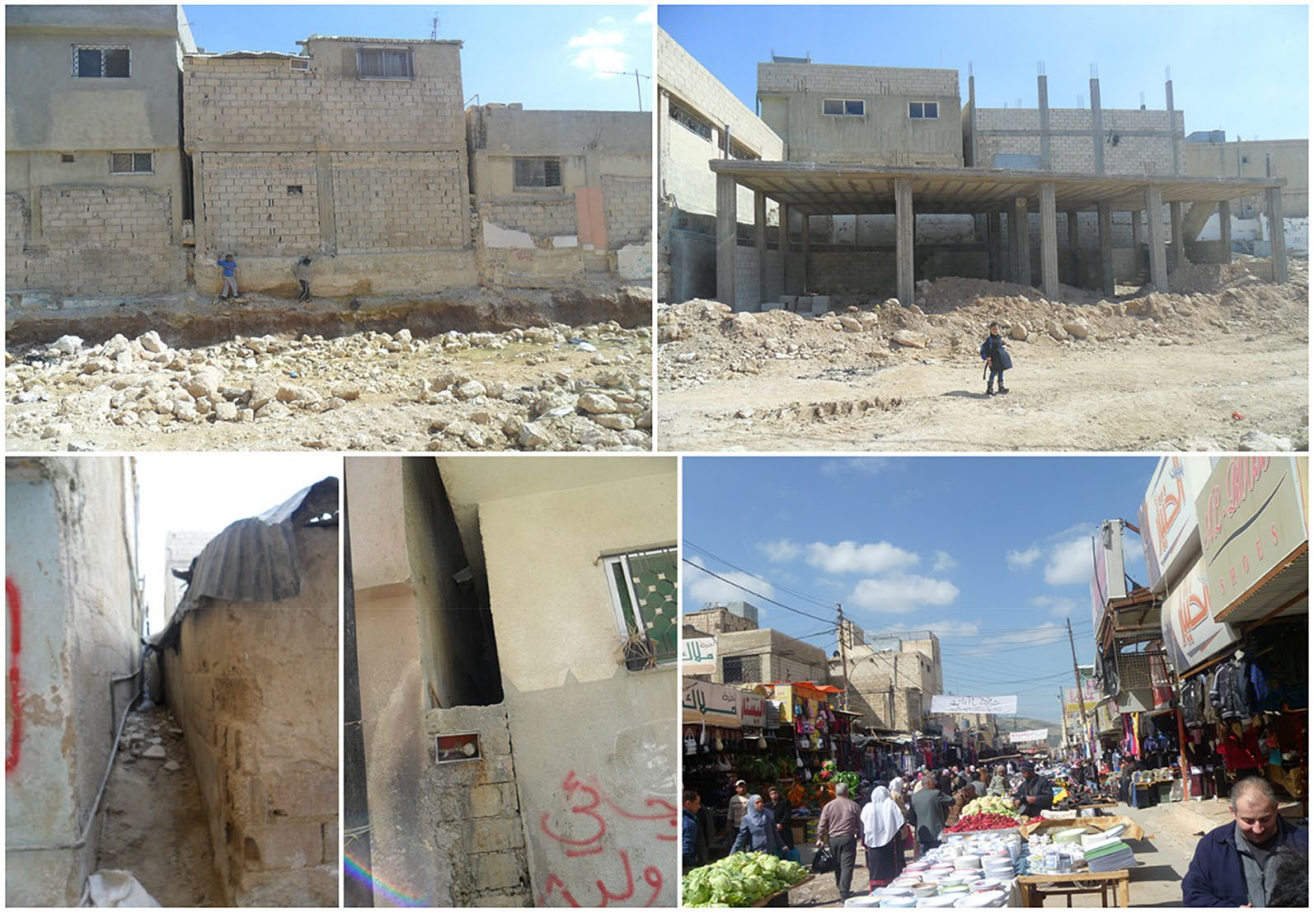
Al Baqa’a camp ( source: Author 2012–2015)

Today, most of the camp’s inhabitants have since built more durable concrete shelters (Figs. 11, 12). The camp is still in need of major upgrading of camp infrastructure, shelter repair and rehabilitation and therefore exacerbate the impact of the extreme climatic conditions. A study on housing conditions in Al Baqa’a camp by Alnsour and Meaton (2014) described the use of inadequate building materials and poor maintenance, leaky roofs and cracked walls, and the lack of windows and adequate ventilation space between building structures. Refugees have to compensate for the poor conditions by using air conditioning in summer time and electric heating during the coldest winter months. However, this is a costly and often inaccessible option for underprivileged refugee households because the energy costs are high relative to their incomes. Interview data revealed that in extreme conditions in summer and winter, 62% of refugees have to walk more than 20 min to reach a shopping area or even children to reach their schools. The experience of walking long distances in extreme high and low temperatures can often discourage refugees from accessing key facilities and services. Findings from the questionnaire supported this challenge by revealing that only 29% of refugees lived near public services and shopping areas. Furthermore, the challenges in walking long distances in extreme temperatures can also limit the social bonding and interaction of refugees with other families and their integration into the refugee camp space. Poverty and high unemployment are significant challenges facing Al Baqa’a camp residents and the camp is ranked third of the ten camps in Jordan in poverty (Fafo Foundation 2013). The use of permanent building structures such as concrete has added a level of climatic comfort for most of the refugees and has led to the creation of spaces for spontaneous day to day practices. These spaces have included areas of economic activity such as the marketplace.

However, space continues to be a contested issue in Al Baqa’a camp and this has adverse impact on how the refugees live and maintain their wellbeing. When questioned about the critical issues affecting the well being of themselves and their families, many responded to the limitations of space which lead to overcrowding and fighting and the need for spaces for community, gathering, traditional sharing and privacy. There is also a gender element to the inadequacy of space as the adverse weather conditions result in female refugees spending long hours inside the shelter. The interviews described how women are suffering from this situation due to the traditional and social practices. Additionally, there are no appropriate outdoor areas for children to play during the extreme temperatures. Therefore, the findings revealed that children are forced to play within insecure outdoor environments or stay indoors for long periods of time.

The challenges in the camp environment and shelter performance as highlighted by the refugees demonstrate the priorities of the humanitarian organizations. The expert interviews noted that the fundamental priority is the protection of refugees. Decisions made on the structural elements of the shelter must consider firstly, management control and the cost of transfer and movement. Climatic conditions and the social contexts are often considered after these have been met, depending on the situation. One expert interviewed with the UNHCR Innovation (2015), explained that producing a new shelter does not mean creating a solution because the flexibility of such a shelter demands changing materials by producing offers from manufacturing companies and meeting diversity of social and cultural aspects by over time. This perspective aligned with other experts from humanitarian organizations who argued for the knowledge of the failed methodology as a solution to creating a process of frequent producing and testing whilst being sensitive to the context and socio-cultural aspects. Despite this, there was an overall appreciation that the logistical factors of transferring the shelters in addition to time and cost were the primary priorities. Some expert interviewees suggest using local materials to deliver shelter longevity. They described how this may allow for adequate shelters that respect the environmental and climatic context whilst achieving social bonding through daily activities. However, there was acknowledgment amongst the experts of the difficulty in applying socio-cultural requirements particularly to communities with diverse cultural and traditional needs.

## Refugees’ socio-spatial practices and self-organization

**Fig. 13:**
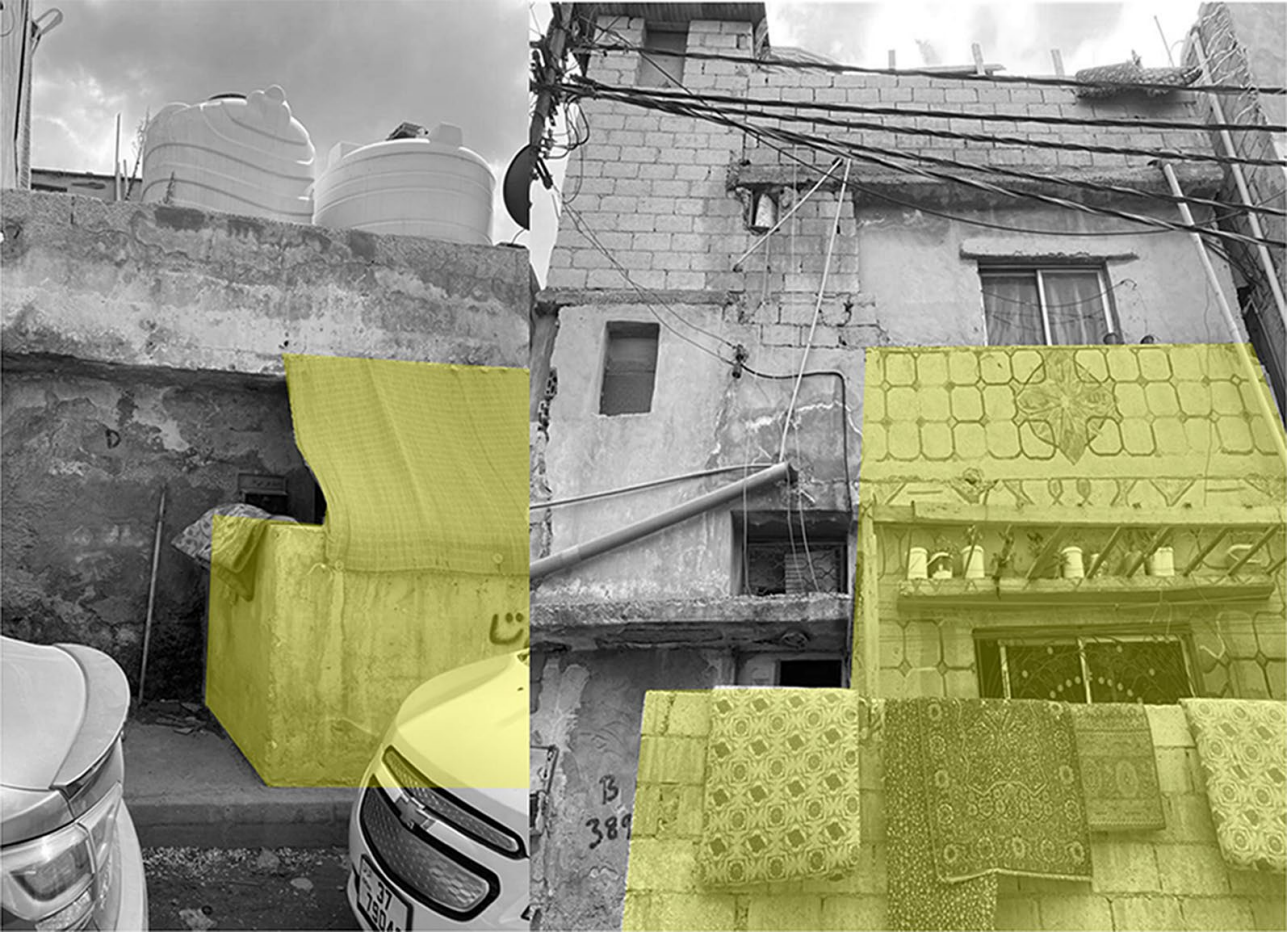
Semi-outdoor private extension (photo taken by Al Baqa’a camp refugee adapted by Author 2021)

Al Baqa’a refugee camp is a place where refugees experience everyday social practices, self-organization and adaptation that influence their spatiality in the camp. These practices have evolved and transformed over the years. The practice of collective meeting and gathering is a significant social practice that has emerged and transformed throughout the years. The meaning and symbolism of gathering has shifted as Al Baqa’a camp transitioned into a space of less temporariness. Regular collective meeting is a social practice that carries multiple meanings and significance for refugees. Refugees in their interviews emphasized how critical, social bonding is to them and the difference it makes in allowing for adaptation and a form of normalcy in the camp space. Although a number of refugees preferred to stay in the same place that they settled in the first moment of arriving which was usually closer to relatives and family instead of moving closer to services in the same camp. Moving away from relatives and family was perceived as moving away from the stability that the refugees are looking for. The spaces for meeting together in the home as collective families is often inadequate and small. Therefore, refugees have self-organized formal and informal spaces dedicated to social and cultural gathering. In-between public spaces are created with refugees meeting outside homes, in streets and at the marketplaces as shown in Fig. 12. However, some elements of the social practices require privacy and therefore such in-between places are not suitable. The formal regulations in Al Baqa’a camp determine how refugees can expand their homes. According to regulations, homes cannot be extended beyond their allocated grid which is between 96 to 120 m^2^. However, the desire and need for private social practices has led to new architectural elements that symbolize a refugee-led spatial organization that challenges and disrupts the formal order. An example of this is shown highlighted in Fig. 13, photos taken by a refugee, of home extensions giving private entrances into the house and providing additional semi-outdoor space. The extensions are built onto the public pavement and some refugees have decorated this area displaying a sense of pride and belonging. These semi-outdoor entrances have become particularly significant for women who have a very limited ability to adapt their clothing as they felt exposed to the outdoors whilst in their own shelters. During the winter these spaces also protect the families from the high winds and from cold during the low temperatures in entering into the main living space, as well as act as shade during the hot summers.

**Fig. 14:**
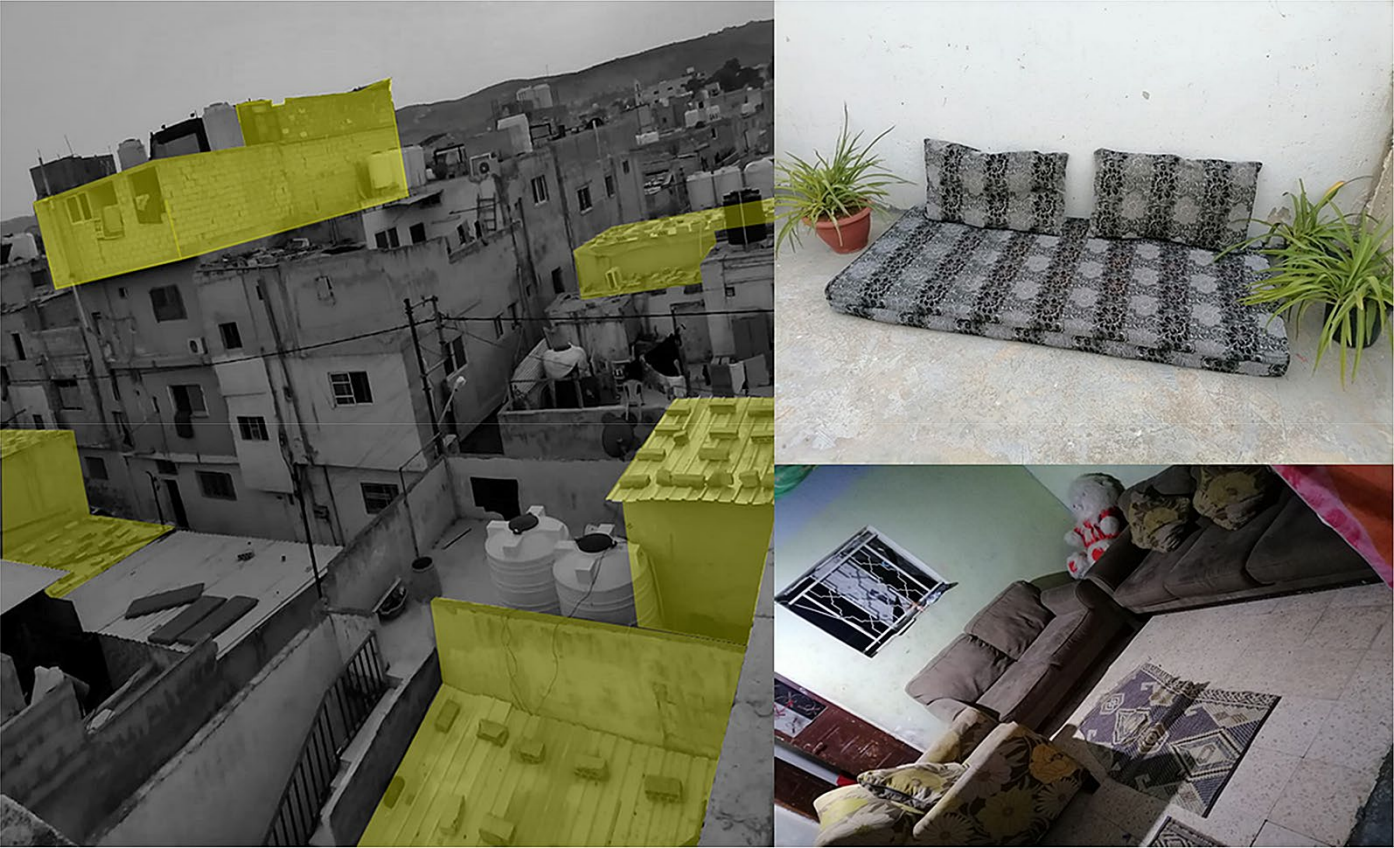
Vertical terrace extension with interior photos of the terrace (photo taken by Al Baqa’a camp refugee adapted by Author 2021)

Over the years, a quasi-permanence has developed in a physical sense associated with the building of permanent concrete homes and infrastructure. However, within this lengthened time of stay, refugees’ practices have become increasingly politicized. Indeed, the vision of political issues has grown stronger within spaces of everyday practice and collective gathering and meeting. Refugees meeting together in the informal in-between spaces share dreams to go back to Palestine. This mutual sharing and collective identity resulted in the need for large, private spaces of gathering for political planning and mobilization. Vertical terraces are designed and built by refugees as spaces intended for everyday social practices of habitual living and for collective politicized practices. An example of this terrace is shown in Fig. 14, a social space with a sofa and indoor plants used by the household and friends and family covered with aggregated metal. Within this space, refugees with common and mutual identity and experiences can meet together comfortably and independently. The increase in collective desire to return home has also resulted in a population increase as families are keen to expand and grow their community for a mass return to Palestine. This has impacted the density of Al Baqa’a camp and the need for housing that supports increasingly large households.

## Emerging lessons learned

This study draws lessons based on the findings and data from the fieldwork, refugee interviews, questionnaires and expert interviews discussed in the previous section. These lessons have the potential to provide guidance to local Governments, humanitarian organizations, Non-Governmental organizations and other diverse stakeholders involved with designing and maintaining refugee camps.

### Perceiving refugee shelters as a process not a product can allow for a more sensitive and integrated approach to shelter design

The findings suggest that humanitarian organizations primarily aim to meet the logistical criteria for shelters with priorities of management of cost and time. Thereby viewing shelter development as a product not as a process and excluding the crucial climatic conditions and socio-cultural aspects. Humanitarian organizations follow policies and obligations of government which lead to standardized solutions and temporary shelters and as a result, they rarely consider factors of shelter performance, materials used, shelter functionality, durability, climatic conditions, building and dismantling and spatial organization, social cohesion and bonding and cultural practices as one process, in their provision of shelters. These solutions lack integration between social practices and environmental challenges and can therefore prove to be inadequate in providing thermal comfort for refugees.

### Finding a balance between climatic conditions, standardization solutions and community needs help to avoid situations of discomfort in the living conditions for refugees

The setup of refugee camps occurs for diverse reasons such as due to man-made disasters or natural disasters and can be created in areas with different climatic conditions. This can also influence the duration of the camp and the community needs and desires. It has been proven that humanitarian organizations tend to provide refugees with standardized shelters that lack the recognition of the specific social needs of the community. However, standardized shelters have been proven inadequate, particularly when the camps go past the point of temporariness such as the Al Baqa’a camp. The refugee led transformations are visible over time as the camp evolves and the refugees begin to use sustainable and natural elements to adapt and live comfortably. Various intermediate conditions such as the prolonging of crisis leads to socio-spatial evolutions and requirements. These social-cultural ways of living should be considered at the outset of refugee camp creation. Consequently, there is no universal agreement of the actual conceptualization of the refugee camp space (Aburamadan et al. 2020). Therefore, a customized and tailored response to the context can give a good balance to avoid situations of discomfort in living conditions.

### Socio-cultural aspects must be considered in the design of refugee camps

A shelter design that considers the cultural and social aspects as well as the thermal performance provides a healthy and dignified living for a population that has lost a lot as a result of being displaced. The findings suggest that to achieve this, there must be an acknowledgment of the “permanence” in activities and needs that may exist within the camp space. Thus, the recognition of the urban socio-cultural requirements that frame a refugee camp as a city or as an incomplete urban formation. Social aspects are a key feature of adapting in different contexts, which could be stimulated by the development process of design that adapts to the local situation. Coping with harsh the climatic conditions for long periods can be extremely challenging on the day to day activities and social practices of refugees. Furthermore, it is difficult to reflect these experiences within universal technical specifications that can lead to a global prototype that is appropriate for diverse contexts. Each camp has different situations and requirements from others. Despite this, considering the differences of social customs and cultural values of societies is an important step which leads to the design of much more successful and sustainable shelters.

### Engaging with refugees as co-creators in designing refugee spaces can provide spaces that cater to the needs of the refugee communities

Refugee families were evidenced to be creating spaces to as coping mechanisms for the harsh climatic conditions as well as spaces for privacy and social interaction. Such spaces proved to be extremely valuable for the refugees during the high temperatures of the summer and high winds and low temperatures in the winter. However, there was significant cost for the refugees associated with this adaptation. This highlights the need to recognize processes of self-organization within the camp space and the role of “human actors as creative beings” (Fuchs 2003). A continuous channel of engagement with the refugees and necessary stakeholders in the process of producing and maintaining the refugee shelters can allow for the prioritization of the user’s needs in relation to adaptation to the climatic conditions of the camp.

### Perceiving refugee shelters as a process not a product can allow for a more sensitive and integrated approach to shelter design

Al Baqa’a camp is an example of how camps can transform and evolve into informal cities over a period of time despite the organization and planning of humanitarian and host organiations. With time the camp changes according to the needs and desires of the refugees. This spontaneous evolution adds to the complexity of how to design and develop refugee camps and therefore warrants careful acknowledgment and consideration.

## Conclusions

The literature review and field work show the challenges that humanitarian organizations and local governments face to offer shelters for refugees in hot dry climates. As shown in the case of the Al Baqa’a camp, the refugees experienced very harsh climatic conditions with inadequate shelter at the start of the camp such as the relief tents. These types of shelter could not provide sufficient thermal comfort. As the camp evolved and the camp infrastructure was upgraded, the refugees self-organized and self-adapted to create places that provided thermal comfort but also considered the socio-cultural aspects of the ways they lived. Despite the upgrade of material to concrete, refugees still needed to self-create spatial elements that provided for meeting and enterprise. This indicates that standardized solutions by humanitarian organizations, local institutions and local governments, to provide shelters as a product are insufficient in meeting refugees’ needs over time. The consequences are costly, because the solution was constructed as prototypes not as a process with the absence of stakeholders’ prioritizations. The social climatic impact must, therefore, be considered in the process of production in terms of social and cultural diversities of societies, that is a limitation to adopt in the process as principles or specification, thus it might give an opportunity for each affected society to contribute through their practices in the field. More and more camps are evolving and transitioning into communities and socio-spatial areas with increased elements of permanence. Therefore, the challenges such as adaptation to climatic elements become increasingly prevalent.

## Data Availability

Data sharing is not applicable to this article as no datasets were generated or analysed during the current study.

## References

[CR1] Aburamadan R, Trillo C, Makore BCN (2020). Designing refugees’ camps: temporary emergency solutions, or contemporary paradigms of incomplete urban citizenship? Insights from Al Za’atari. City Territ Archit.

[CR2] Agier M (2002). Between war and city. towards an urban anthropology of refugee camps. Ethnography.

[CR3] Agier M (2008). On the margins of the world: the refugee experience today.

[CR4] Albadra D, Vellei M, Coley D (2017). Thermal comfort in desert refugee camps. Build Environ.

[CR5] Albadra D, Elamin Z, Adeyeye K, Polychronaki E, Coley DA, Holley J, Copping A (2021). Participatory design in refugee camps: comparison of different methods and visualization tools. Build Res Inform.

[CR6] Alnsour J, Meaton J (2014). Housing conditions in Palestinian refugee camps, Jordan. Cities.

[CR7] Ashmore J, Babister E, Corsellis T (2003). Diversity and adaptation of shelters in transitional settlements for IDPs in Afghanistan. Disasters.

[CR8] Attia S (2014). Assessing the thermal performance of Bedouin tents in hot climates.

[CR9] Brown B, Altman I, Werner C (2012). Place attachment. Int Encycl Hous Home.

[CR10] Brun C (2001). Reterritorilizing the relationship between people and place in refugee studies. Geogr Ann.

[CR11] Certeau Md, Giard L, Mayol P (1998). The practice of everyday life: living and cooking.

[CR13] Corsellis T, Vitale A (2005). Transitional settlement: displaced populations.

[CR14] Corsellis T, Vitale A (2008). Transitional settlement and reconstruction after natural disaster.

[CR17] Fábos A, Kibreab G (2009). Urban refugees: introduction.

[CR900] Fafo Foundation (2013) Progress, challenges, diversity: insights into the socio-economic conditions of Palestinian refugees in Jordan. Fafo-report 2013:42. https://www.unrwa.org/resources/reports/insights-socio-economic-conditions-palestinian-refugees-jordan

[CR18] Flyvbjerg B (2006). Five misunderstandings about case-study research. Qual Inq.

[CR901] Fuchs C (2003). Structuration theory and self-organization. Syst Pract Action Res.

[CR19] Hart J, Paszkiewicz N, Albadra D (2018). Shelter as home? Syrian homemaking in Jordanian refugee camps. Human Organ.

[CR21] Herz M (2012). From camp to city: refugee camps of the Western Sahara.

[CR22] Institute for Palestine Studies (2021) Palestinian journeys, timeline. https://www.paljourneys.org/en. Accessed 20 Mar 2021

[CR23] Johansson E, Ouahrani D, Shaker Al-Asir H (2009). Climate conscious architecture and urban design in Jordan-towards energy efficient buildings and improved urban microclimate. Report.

[CR25] Kennedy J, Ashmore J, Babister E (2008). The meaning of ‘build back better’: evidence from post-tsunami Aceh and Sri Lanka. J Contingencies Crisis Manag.

[CR26] Klansek T, Coley DA, Paszkiewicz N (2020). Analysing experiences and issues in self-built shelters in Bangladesh using transdisciplinary approach. J Hous Built Environ.

[CR27] Knox P, Mayer H (2013). Small town sustainability: Economic, social, and environmental innovation.

[CR28] Ledwith A (2014). Zaatari: the instant city.

[CR29] Lintelo DT, Lakshman R, Mansour W (2018). Wellbeing and protracted urban displacement: refugees and hosts in Jordan and Lebanon.

[CR30] Livingston M, Bailey N, Kearns A (2008). People’s attachment to place: the influence of neighbourhood deprivation.

[CR31] Lombard M (2014). Constructing ordinary places: place-making in urban informal settlements in Mexico. Prog Plan.

[CR32] Mah KW, Rivers PL (2016). Refugee housing without exception. Space Cult.

[CR33] Manfield P (2000). A comparative study of temporary shelters used in cold climates.

[CR34] Manfield P, Ashmore J, Corsellis T (2004). Design of humanitarian tents for use in cold climates. Building Research & Information.

[CR48] Maqusi S (2017). ‘Space of refuge’: negotiating space with refugees inside the Palestinian camp. Humanities.

[CR36] Paszkiewicz N, Fosas D (2019). Reclaiming refugee agency and its implications for shelter design in refugee camps.

[CR37] Sampson R, Gifford SM (2010). Place-making, settlement and well-being: the therapeutic landscapes of recently arrived youth with refugee backgrounds. Health Place.

[CR38] Scannell L, Gifford R (2010). Defining place attachment: a tripartite organizing framework. J Environ Psychol.

[CR39] Schmeidl S (2002). (Human) security dilemmas: long-term implications of the Afghan refugee crisis. Third World Q.

[CR41] Stevenson A, Sutton R (2011). There’s no place like a refugee camp? Urban planning and participation in the camp context.

[CR902] UNHCR Innovation (2015) Beyond technology: innovation at UNHCR 2015. https://www.unhcr.org/innovation/wp-content/uploads/2016/10/Innovation_at_UNHCR_2015.pdf

[CR42] UNHCR (2019). Global trends 2019: forced displacement in 2019.

[CR44] UNHCR (2020) 1 per cent of humanity displaced: UNHCR Global Trends report. https://www.unhcr.org/uk/news/press/2020/6/5ee9db2e4/1-cent-humanity-displaced-unhcr-global-trends-report.html. Accessed 18 June 2020

[CR45] UNRWA (2020). UNRWA where we work, Jordan.

[CR46] UNWRA (2013). Unrwa in figures—July 2013.

